# Development and implementation of an ultralow-dose CT protocol for the assessment of cerebrospinal shunts in adult hydrocephalus

**DOI:** 10.1186/s41747-021-00222-4

**Published:** 2021-06-28

**Authors:** David J. Ryan, Richard G. Kavanagh, Stella Joyce, Mika O’Callaghan Maher, Niamh Moore, Aisling McMahon, Deirdre Hussey, Michael G. J. O’Sullivan, Gerald Wyse, Noel Fanning, Owen J. O’Connor, Michael M. Maher

**Affiliations:** 1 Department of Radiology, Cork University Hospital, Wilton, Cork city, T12 DC4A Ireland; 2 Department of Radiography, School of Medicine, Brookfield Health Sciences Complex, University College Cork, Cork, T12 AK54 Ireland; 3 Department of Medical Physics, Cork University Hospital, Wilton, Cork city, T12 DC4A Ireland; 4 Department of Neurosurgery, Cork University Hospital, Wilton, Cork city, T12 DC4A Ireland; 5 School of Medicine, University College Cork, Cork, Ireland

**Keywords:** Adult, Cerebrospinal fluid shunts, Hydrocephalus, Radiation dosage, Tomography (x-ray, computed)

## Abstract

**Background:**

Cerebrospinal fluid shunts in the treatment of hydrocephalus, although associated with clinical benefit, have a high failure rate with repeat computed tomography (CT) imaging resulting in a substantial cumulative radiation dose. Therefore, we sought to develop a whole-body ultralow-dose (ULD) CT protocol for the investigation of shunt malfunction and compare it with the reference standard, plain radiographic shunt series (PRSS).

**Methods:**

Following ethical approval, using an anthropomorphic phantom and a human cadaveric ventriculoperitoneal shunt model, a whole-body ULD-CT protocol incorporating two iterative reconstruction (IR) algorithms, pure IR and hybrid IR, including 60% filtered back projection and 40% IR was evaluated in 18 adult patients post new shunt implantation or where shunt malfunction was suspected. Effective dose (ED) and image quality were analysed.

**Results:**

ULD-CT permitted a 36% radiation dose reduction (median ED 0.16 mSv, range 0.07–0.17, *versus* 0.25 mSv (0.06–1.69 mSv) for PRSS (*p* = 0.002). Shunt visualisation in the thoracoabdominal cavities was improved with ULD-CT with pure IR (*p* = 0.004 and *p* = 0.031, respectively) and, in contrast to PRSS, permitted visualisation of the entire shunt course (*p* < 0.001), the distal shunt entry point and location of the shunt tip in all cases. For shunt complications, ULD-CT had a perfect specificity. False positives (3/22, 13.6%) were observed with PRSS.

**Conclusions:**

At a significantly reduced radiation dose, whole body ULD-CT with pure IR demonstrated diagnostic superiority over PRSS in the evaluation of cerebrospinal fluid shunt malfunction.

**Supplementary Information:**

The online version contains supplementary material available at 10.1186/s41747-021-00222-4.

## Key points

- Whole-body ultralow-dose computed tomography (CT) with pure iterative reconstruction demonstrates diagnostic superiority over plain radiographic shunt series in the evaluation of cerebrospinal fluid shunt malfunction.
- With a median effective dose of 0.16 mSv, representing a 36% radiation dose reduction relative to plain radiographic shunt series, ultralow-dose CT may permit a reduction in the cumulative radiation dose received by patients evaluated for cerebrospinal fluid shunt malfunction.
- By enabling assessment of the brain and entire shunt with one imaging modality, this novel protocol may help establish CT as the single point of care in adult and paediatric patients with suspected shunt malfunction.

## Background

Hydrocephalus is a disorder of abnormal cerebrospinal fluid (CSF) dynamics. Disturbances in formation, flow or absorption result in the accumulation of excess CSF within the calvarium [1]. There are multiple aetiologies for hydrocephalus which can be broadly classified as being congenital or acquired [1]. Surgical placement of a CSF shunt is the most commonly performed therapeutic procedure for hydrocephalus. Shunts though associated with significant clinical benefit remain one of the most failure-prone of all implanted medical devices accounting for approximately $2 billion in annual healthcare expenditure in the USA [2]. In paediatric patients, shunt failure encompassing obstruction, infection and mechanical shunt failure occurs in 30–40% at 1 year and approximately 50% at 2 years [3].

In suspected shunt failure, brain computed tomography (CT) is initially performed to assess ventricular size, identify features suggestive of raised intracranial pressure and evaluate the location and integrity of the intra-cranial portion of the shunt. To evaluate extracranial shunt integrity, the standard first line investigation is a shunt series comprising plain radiographs of the entire shunt course [4]. Shunt series, though accepted as the current gold standard investigation, is not a low-dose examination with reported effective dose (ED) of 1.57 ± 0.60 mSv (mean ± standard deviation) per study [5]. Given the high failure rate of inserted shunts and consequent need for repeat imaging, the cumulative radiation dose received over an individual’s lifetime can be considerable [6, 7]. This is clearly of concern in paediatric patients where exposure to radiation in childhood is associated with an increased lifetime cancer risk [8]. Therefore, alternative imaging strategies are required.

Low-dose whole-body CT has been investigated for this purpose and has been shown to possess high sensitivity and specificity for the detection of shunt complications in both swine and rabbit models [9–11]. Excitingly, this was achieved at radiation doses lower than plain radiographic shunt series (PRSS) [11]. Similarly, in humans, low-dose whole-body CT allows more accurate visualisation of shunt position than PRSS without increasing the associated radiation exposure [12]. However, although promising, the achieved effective dose of 1.9 mSv with low-dose CT theoretically may still result in a significant cumulative radiation exposure due to multiple examinations.

Therefore, we endeavoured to develop and implement a whole-body ultralow-dose (ULD) CT protocol that at a minimum was diagnostically equivalent to PRSS in the evaluation of shunt integrity and related complications. As reductions in radiation dose result in increased image noise and poorer image quality, we sought to utilise iterative reconstruction (IR) algorithms which are well-known to preserve image quality at low radiation doses by incorporating statistical information including photon statistics and electronic acquisition noise. The IR algorithms, hybrid IR comprising 60% filtered back projection (FBP) and 40% adaptive statistical iterative reconstruction (ASIR) and the pure IR algorithm, model-based iterative reconstruction (MBIR), were compared in this study. As all CT manufacturers have incorporated their own vendor-specific iterative reconstruction algorithms into commercially available CT scanners, the low-dose CT protocol described herein should be achievable in all centres.

## Methods

**Fig. 1:**
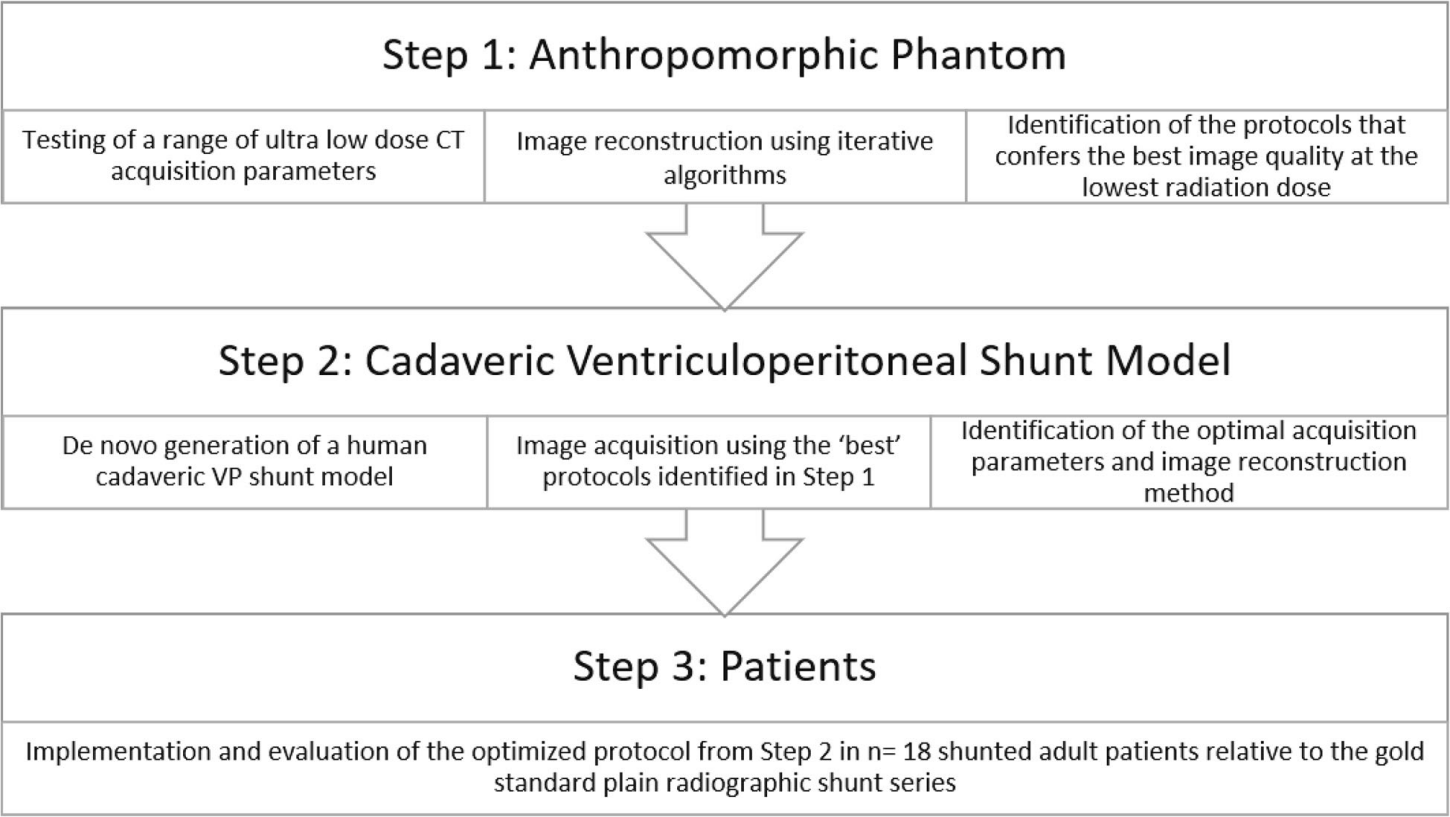
Study flow diagram. *VP* Ventriculoperitoneal

Following obtaining institutional ethical board approval, the study was split into two components: CT protocol development and implementation of the newly designed protocol. Protocol development was performed prior to patient recruitment. The methodological steps are summarised in Fig. 1.

### CT protocol development

Initial development of the ULD CT protocol was performed using a commercially available life-sized anthropomorphic phantom (Kyoto Kagaku Co., Kyoto, Japan). A range of scanning parameters were chosen to evaluate with the resulting radiation doses recorded. The phantom-based protocols which conferred the best subjective image quality at the lowest effective dose were then tested in a newly developed human cadaveric ventriculoperitoneal shunt model.

To create the cadaveric model, a shunt system (CODMAN® BACTISEAL®, Johnson & Johnson, Raynham, MA, USA) was inserted by a neurosurgical resident. Briefly, a right parietal burr hole was created, and the dura was incised. A ventriculostomy catheter was inserted into the lateral ventricle. Distally, following incising the abdominal skin, a small peritoneal opening was made in the right upper quadrant. The shunt tubing was tunnelled distally and inserted into the peritoneal cavity. The proximal tubing and ventriculostomy catheter were attached to a one-way valve which was secured in a fashioned subcutaneous pocket. Wound closure was performed using non-absorbable sutures.

For each protocol/acquisition setting in the cadaveric model, images were reconstructed using hybrid IR and pure IR, and objective image noise was calculated by placing regions of interest (ROIs) in pre-defined anatomical substructures at neck, thoracic and abdominal levels (see below “Objective image quality analysis” section). Difference in means at each anatomical level was assessed using paired sample *t*-tests. Additionally, the dose length product (DLP) associated with each protocol was recorded. Final protocol selection was dependent on the attainment of diagnostically acceptable image quality at the lowest achievable radiation dose.

### Patient selection

Non-sequential convenience sampling was used for patient recruitment in this prospective single-centre cohort study. Any adult patient who had undergone new shunt implantation or in whom shunt malfunction was suspected was eligible for study inclusion. Exclusion criteria included shunted paediatric and pregnant patients. At our institution post new shunt insertion, baseline PRSS is performed to evaluate catheter position and to assess for any immediate post-procedural complications. Written informed consent was obtained from the participating patient whenever possible. In specific cases, including those patients lacking capacity, consent was acquired from guardians. All patients proceeded to PRSS, standard dose unenhanced CT brain and ULD CT.

### Imaging technique

Images were acquired using a 64-slice multidetector CT scanner (Discovery CT 750HD; GE Healthcare, GE Medical Systems, Milwaukee, WI, USA). The final protocol parameters were 100 kVp, 10 mA and pitch 1.375. No contrast material was administered. The CT acquisition parameters are listed in Table 1. The whole-body ULD scan range extended from the vertex to the proximal femurs. A reduced range ULD CT excluding the brain extending caudally from the skull base was performed in selected patients. As per standard departmental policy, all imaging reports were immediately available for review by the referring physician. Images were reconstructed with hybrid IR comprising 60% FBP and 40% ASIR (GE Healthcare, WI, USA) as per standard departmental procedures or with the pure IR algorithm, MBIR (VEO, GE Healthcare, Milwaukee, WI, USA). Calibration of the CT unit was performed once weekly in accordance with manufacturer guidelines and local departmental quality assurance protocols. For the PRSS, two digital radiographic units were used in this study, the Digital Diagnost 3.1 system (Philips, Eindhoven, The Netherlands) and the DRX Evolution Plus system (Carestream Health Inc, Rochester, NY, USA). The radiographs performed as part of the PRSS were determined by the type of implanted shunt. Standard PRSS comprised a lateral skull and neck radiogram, a posteroanterior chest radiogram and a supine abdominal radiogram.

**Table 1:** Computed Tomography acquisition parameters

Acquisition parameters	
Detector rows	64
Scan mode	Helical
kVp	100
mA	10
Displayed field of view (cm)	308
Filter type	Body
Matrix	512 × 512
In-plane resolution (mm)	0.601
Reconstructed slice thickness (mm)	3
Focal spot size (mm)	0.7
Revolution time (milliseconds)	400
Pitch factor	1.375

*kVp* Kilovoltage peak, *mA* Milliampere

### Dose measurements

All dose calculations were performed by a senior departmental medical physicist. The Imaging Performance and Assessment in CT dosimetry calculator (ImPACT version 0.99x, London, UK) was used to calculate effective dose (ED) for ULD CT. Parameters extracted from the DICOM dataset within the Impax 6.5.3 Picture Archiving and Communication System (Agfa Healthcare, Morsel, Belgium) for the dose calculation were DLP, kVp, mA, rotation time, and pitch. PRSS-associated EDs were calculated using the PCXMC 2.0 software (STUK, Helsinki, Finland). Data included in the dose calculations were half value layer for the room, kVp, dose area product, added filtration (when used), and focus to detector distance which was included in the DICOM data.

### Objective image quality analysis

ULD CT images reconstructed with pure IR and hybrid IR were evaluated for objective image quality. Analysis was performed on a dedicated workstation (Advantage Workstation VolumeShare 2, Version 4.4, GE Medical Systems, Milwaukee, WI, USA). Image quality was assessed in consensus by two radiologists with 6- and 7-years radiology experience, respectively. Ten-mm circular ROIs were placed in pre-defined anatomical sub-structures of the neck, thoracic, abdominal and pelvic cavities (Supplementary Table S1). In those subjects who proceeded to ULD CT brain imaging, ROIs were also placed intracranially. Efforts were made to place the ROI in area as homogenous as possible, away from blood vessels and fat planes.

To evaluate objective noise in conventional and ULD thoracic CT, 10-mm ROIs were placed in the aorta, subcutaneous fat, paraspinal muscle and lung at the level of the carina, greatest transverse cardiac diameter and dome of the diaphragm. In the upper abdomen, 10-mm ROIs were placed in the left, right and central liver at the level of the porta hepatis. For direct comparison of conventional and ULD brain CT, 10-mm ROIs were placed on the same axial slice in the caudate nucleus, posterior limb of the internal capsule and frontal horn of the lateral ventricle supratentorially and in the deep cerebellar white matter and cerebellar cortical grey matter infratentorially [13]. In all studies, the mean attenuation value ($\overline{X}$) within the ROI and the standard deviation (*σ*) represented the signal and noise level, respectively. Triplicate measurements were taken to minimise error. The signal-to-noise ratio (SNR) within each ROI was calculated by dividing the mean HU by its standard deviation. Contrast-to-noise ratios in the supratentorial and infratentorial compartments was calculated for ROI pairs using the following formula [13]:

$$ CNR=\frac{\overline{X1}\kern0.5em -\overline{X2}}{\sqrt{\sigma {1}^2+\sigma {2}^2}} $$

Objective noise indices were statistically compared at each measured level for both data sets.

### Subjective image quality analysis

Subjective image quality was assessed in consensus by 2 radiologists, with 13- and 22-years radiology experience, respectively. Research reads were blinded to all previous radiological results and clinical information. All PRSS and ULD CT-Pure IR images were reviewed with a high-resolution monitor (3 megapixels), using soft-tissue window settings only (window width 400 HU, window level 40 HU). Initial subjective analysis of acquired images focused on two parameters: the quality of two-dimensional shunt visualisation at each anatomical level for PRSS and ULD CT using pure IR and the visualisation quality of tissues and/or organs contiguous with the shunt on ULD CT using pure IR. Both parameters were scored using a 5-point Likert scale with the following rating options: 1, not seen; 2, suboptimal; 3, satisfactory; 4, good; and 5, excellent.

PRSS and ULD CT-Pure IR images were further assessed using a binary scale (1, yes; 2, no) for (1) demonstration of exact shunt entry point to peritoneum/pleura/atrium, (2) clear demonstration of shunt tip, (3) precise identification of anatomic location of shunt tip, (4) visualisation of entire imaged shunt course, and (5) demonstration of shunt complications (including shunt fracture/discontinuity, shunt kink, or organ damage arising from the shunt).

### Statistical analysis

Data were exported from Microsoft Office Excel (Microsoft Corporation, CA, USA) into GraphPad Prism Version 7.1 (GraphPad Software Incorporated, San Diego, USA) for statistical analysis. For continuous variables, Shapiro-Wilk normality testing directed the use of parametric or non-parametric paired analyses, respectively. Mean differences in objective image noise between hybrid IR and pure IR reconstructed images in the cadaveric model and in patients were assessed using paired sample t-tests. Wilcoxon matched pairs signed rank tests were used to assess the difference in radiation doses between PRSS and ULD CT and for comparative assessment of median Likert scores for subjective image quality. Testing the difference between the binary outcomes for clinical parameters was performed using Fisher exact test. Wilcoxon matched-pairs signed rank test and paired samples *t*-test were used to compare image noise and SNR or contrast-to-noise ratio between conventional dose and ULD CT brain imaging reconstructed with pure IR, respectively. A comparison of mean image noise and SNR between standard and ULD thoracic CT reconstructed with pure IR was performed using paired sample *t*-tests. As image analysis was performed in consensus between readers, we did not assess interobserver variability. Differences with a *p-*value < 0.05 were considered significant. Data are presented as mean ± standard deviation unless otherwise specified in the text.

## Results

### Phantom- and cadaver-based CT protocol development

**Fig. 2:**
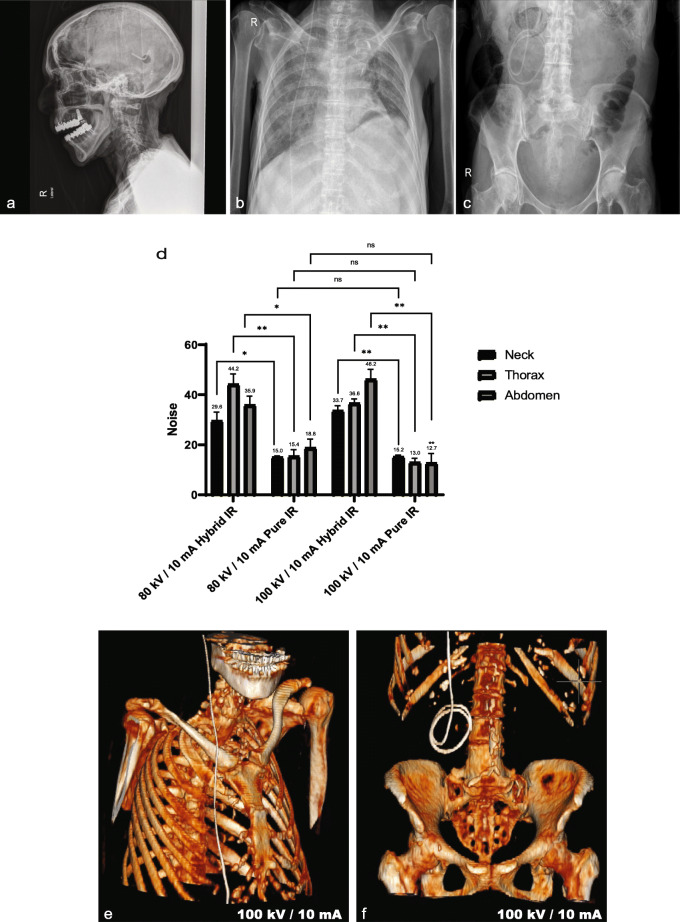
Phantom- and cadaver-based computed tomography protocol development. Plain radiographic shunt series confirming appropriate positioning of the *de novo* inserted ventriculoperitoneal shunt (**a**–**c**). Objective image noise values for the neck, thorax, and abdomen using two kVp/mA settings (80/10 and 100/10) reconstructed using hybrid IR and pure IR (**d**). Data represent mean and standard deviation. Difference in means at each anatomical level was assessed using paired sample *t*-tests. For comparisons of hybrid IR and pure IR at 80/10 (neck: * *p* = 0.019; thorax: **p = 0.003; abdomen: *p = 0.013) and 100/10 (neck: ** *p* = 0.002; thorax: **p = 0.004; abdomen: **p = 0.007). There was no significant difference (ns) in image noise between pure IR images acquired at 80/10 or 100/10. (**e**) Three-dimensional reconstruction demonstrating the ventriculoperitoneal shunt at the final protocol setting 100/10

The newly developed cadaveric shunt model was fit for purpose with PRSS confirming appropriate shunt position (Fig. 2a–c). Whole-body CT with acquisition parameters—80 kVp and 10 mA (80/10) or 100 kVp and 10 mA (100/10)—were chosen to evaluate in the cadaveric system as they were associated with the lowest effective doses (0.11 and 0.15 mSv, respectively) in the anthropomorphic phantom.

At all levels, in both protocols (80/10 and 100/10), pure IR was superior to hybrid IR with a significant reduction in image noise demonstrated: 80/10 (neck *p* = 0.019, thorax *p* = 0.003, abdomen *p* = 0.013) and 100/10 (neck p = 0.002, thorax *p* = 0.004, abdomen *p* = 0.007) (Fig. 2d). There was no significant difference in image noise between pure IR images acquired at 80/10 or 100/10 although the trend suggested that 100/10 may be superior (neck *p* = 0.747, thorax *p* = 0.428, abdomen *p* = 0.051) (Fig. 2d). DLPs associated with 80/10 and 100/10 were 5.7 and 10.7 mGy*cm, respectively. Subjective shunt visualisation was superior using 100/10 with clear visualisation of the shunt course, peritoneal entry point and location of the distal catheter tip (Fig. 2e, f). Therefore, we proceeded to test this ULD protocol in patients.

### Implementation of the newly designed protocol

#### Patient characteristics

Eighteen patients, 11 females (61.1%) and 7 males (38.9%), with a median age of 49 years (range 17–87) were prospectively enrolled. Normal pressure hydrocephalus (7/18, 38.9%) and congenital hydrocephalus (5/18, 27.8%) accounted for two thirds of cases. The remainder were secondary to neoplasm (4/18, 22.2%), benign intracranial hypertension (1/18, 5.6%) and neonatal meningitis (1/18, 5.6%). Imaging was performed for baseline postoperative evaluation of newly inserted shunts and investigation of shunt dysfunction in 5/18 (38.5%) and 13/18 (61.5%) patients, respectively. All patients underwent PRSS and standard dose CT brain. Whole-body ULD CT was performed in 7/18 (38.9%). The remainder underwent reduced range ULD-CT. All imaging investigations were conducted on the same day in 16/18 (88.9%) and within 5 days in the remainder.

Twenty-two shunts were available for evaluation. A single shunt had been placed in the majority (14/18, 77.8%). Four patients (22.2%) had two implanted shunts. Ventriculoperitoneal shunting was most commonly performed (19/22, 86.4%). Ventriculoatrial, ventriculopleural and lumboperitoneal shunts were each seen in a single patient (3/22, 13.6%). Four *de novo* ventriculoperitoneal shunts and one ventriculopleural shunt were placed in 5/18 patients (27.8%)*.*

In all patients evaluated for shunt dysfunction (13/18 [61.5%]), brain imaging was stable with no interval change in ventricular size. In addition, no radiological features to suggest raised intra-cranial pressure or an alternative diagnosis were seen. A possible kink in the shunt tubing was observed in 3/18 (16.7%) on PRSS. A fracture of old shunt tubing which remained *in situ* was seen in 4/18 (22.2%).

#### Radiation dose

**Fig. 3:**
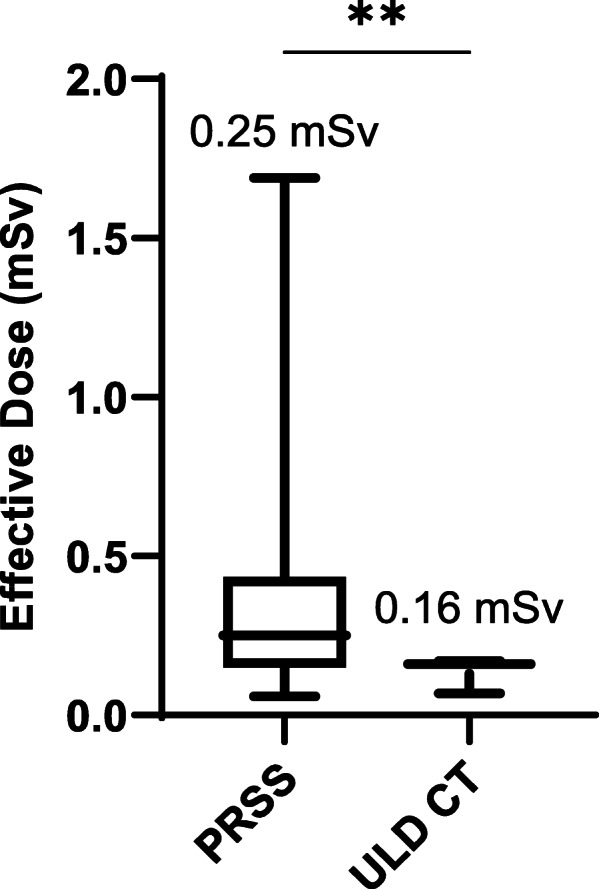
Radiation dose reduction with ultralow-dose computed tomography (ULD CT) using pure iterative reconstruction. Comparison of the effective dose (mSv) between plain radiographic shunt series (PRSS) and ULD CT using a Wilcoxon matched-pairs signed rank test (** *p* = 0.002). Data represent the median, interquartile range, minimum and maximum values

All patients considered, the median DLP for ULD CT was 9.56 mGy*cm (range 2.3–12.2 mGy*cm) conferring a median ED of 0.16 mSv (range 0.07–0.17 mSv). The median EDs for whole-body and reduced range ULD CT were 0.16 mSv (range 0.15–0.17) and 0.14 mSv (range 0.07–0.16) respectively. Individual effective dose for the ventriculoatrial, ventriculoperitoneal, and lumboperitoneal shunts were 0.27 mSv (PRSS) and 0.16 mSv (ULD CT), 0.20 mSv (PRSS) and 0.15 mSv (ULD CT), and 0.06 mSv (PRSS) and 0.069 mSv (ULD CT), respectively. Median dose area product readings for the PRSS were as follows: skull radiograph 4.18 dGy*cm^2^ (range 1.70–9.36), chest radiograph 0.91 dGy*cm^2^ (range 0.36–5.22), and abdominal radiograph 7.49 dGy*cm^2^ (range 1.07–93.3). The median total dose area product was 16.63 dGy*cm^2^ (range 4.45–98.48), conferring an ED of 0.25 mSv (range 0.06–1.69) (Fig. 3). Therefore, all patients considered, the ULD CT protocol resulted into a 36% reduction in ED (*p* = 0.002, Wilcoxon matched-pairs signed rank test).

#### Image quality assessment

**Fig. 4:**
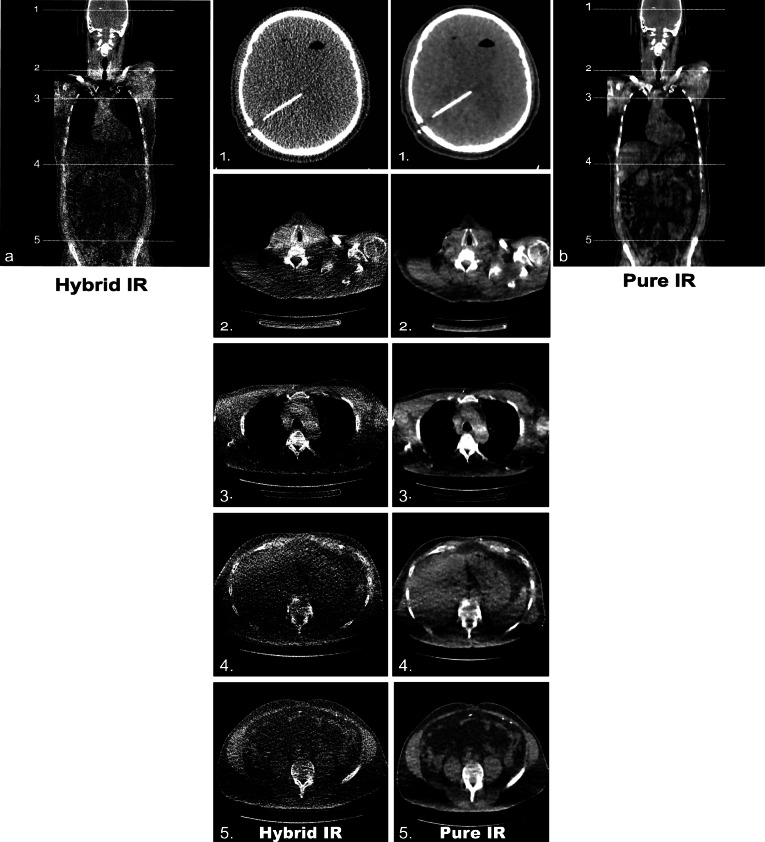
Image quality comparison for ULD CT: hybrid *versus* pure IR. ULD CT coronal (3-mm thickness) images reconstructed with hybrid IR (**a**) and pure IR (**b**). Representative axial (3-mm thickness) images at five anatomical levels (1, head; 2, neck; 3, thorax; 4, abdomen; 5, pelvis) are shown for hybrid IR (left) and MBIR (right). Images were visualised using soft-tissue window settings (window width 400 HU; window level 40 HU). *ASIR* Adaptive statistical iterative reconstruction, *CT* Computed tomography, *FBP* Filtered back projection, *IR* Iterative reconstruction, *MBIR* Model-based iterative reconstruction, *ULD* Ultralow dose

At all anatomical levels, objective noise was significantly lower on ULD CT images reconstructed with pure IR compared with hybrid IR (*p* < 0.00, paired sample *t*-test) (Table 2). Noise reductions of 85.3%, 83.3%, 89.7%, 87.3%, and 89.4% were demonstrated for the head, neck, thoracic, abdominal and pelvic cavities, respectively (Fig. 4 and Table 2). Therefore, pure IR was used for all further analyses.

**Table 2:** Objective image noise: pure *versus* hybrid iterative reconstruction

Anatomical cavity	Mean IN-pure IR	Mean IN-hybrid IR	Noise reduction	***p-***value
Head	7.81 ± 3.06	52.94 ± 14.46	85.3%	*p* < 0.0001
Neck	9.52 ± 9.98	57.13 ± 44.65	83.3%	*p* < 0.0001
Thorax	8.69 ± 4.95	84.24 ± 38.52	89.7%	*p* < 0.0001
Abdomen	12.5 ± 7.18	98.59 ± 43.29	87.3%	*p* < 0.0001
Pelvis	9.23 ± 5.55	87.15 ± 48.1	89.4%	*p* < 0.0001

*IN* Image noise, *IR* Iterative reconstruction

Data represent mean ± standard deviation. Difference in means was assessed using a paired sample t-test. A *p*-value < 0.05 was considered statistically significant

Next, the quality of two-dimensional shunt visualisation at each level was assessed. In the head and neck, PRSS and ULD CT-Pure IR were comparable without significant difference observed (*p* > 0.999 and *p* = 0.125 respectively, Wilcoxon matched-pairs signed rank test). In the thorax and abdomen, however, visualisation quality was significantly better with ULD CT-Pure IR (*p* = 0.004 and *p* = 0.031, respectively, Wilcoxon matched-pairs signed rank test) (Supplementary Fig. S1). The ability to visualise tissues and/or organs contiguous with the shunt on ULD CT images reconstructed with pure IR was rated as satisfactory to excellent with median Likert scores of 3, 5, 4, and 3.5 for the head, neck, thoracic, and abdominal cavities, respectively.

#### Clinical parameter assessment and evaluation for shunt complications

Concordant with the image quality findings, there was a significant difference in the ability to visualise the entire shunt course being achieved in 22/22 (100%) with ULD CT reconstructed with pure IR and 8/22 (36.4%) with PRSS (*p* < 0.001, Fisher exact test) (Table 3). Analysis by shunt subtype demonstrated complete visualisation in 7/19 (36.8%) and 1/1 (100%) for ventriculoperitoneal and ventriculopleural shunts, respectively, with PRSS. The shunt course of ventriculoatrial [1/1] and lumboperitoneal [1/1] shunts could not be entirely visualised with PRSS.

**Table 3:** Clinical parameters of the studied subjects

Parameter	ULD CT-Pure IR	PRSS	***p***-value
Demonstration of exact shunt entry point to peritoneum/pleura/atrium	22/22 (100%)	Not testable	-
Clear demonstration of shunt tip	22/22 (100%)	21/22 (95.5%)	*p* > 0.009
Precise identification of anatomic location of shunt tip	22/22 (100%)	Not testable	-
Visualisation of entire imaged shunt course	22/22 (100%)	8/22 (36.4%)	*p* < 0.001
Demonstration of shunt complications	Not testable *	Not testable *	**-**

PRSS and ULD CT-Pure IR images were compared using a binary scale (1, yes; 2, no) for the included parameters. Contingency squares were generated for each variable, and Fisher’s exact test was performed. A *p*-value < 0.05 was considered statistically significant

*There were no shunt-related complications in this study

**Fig. 5:**
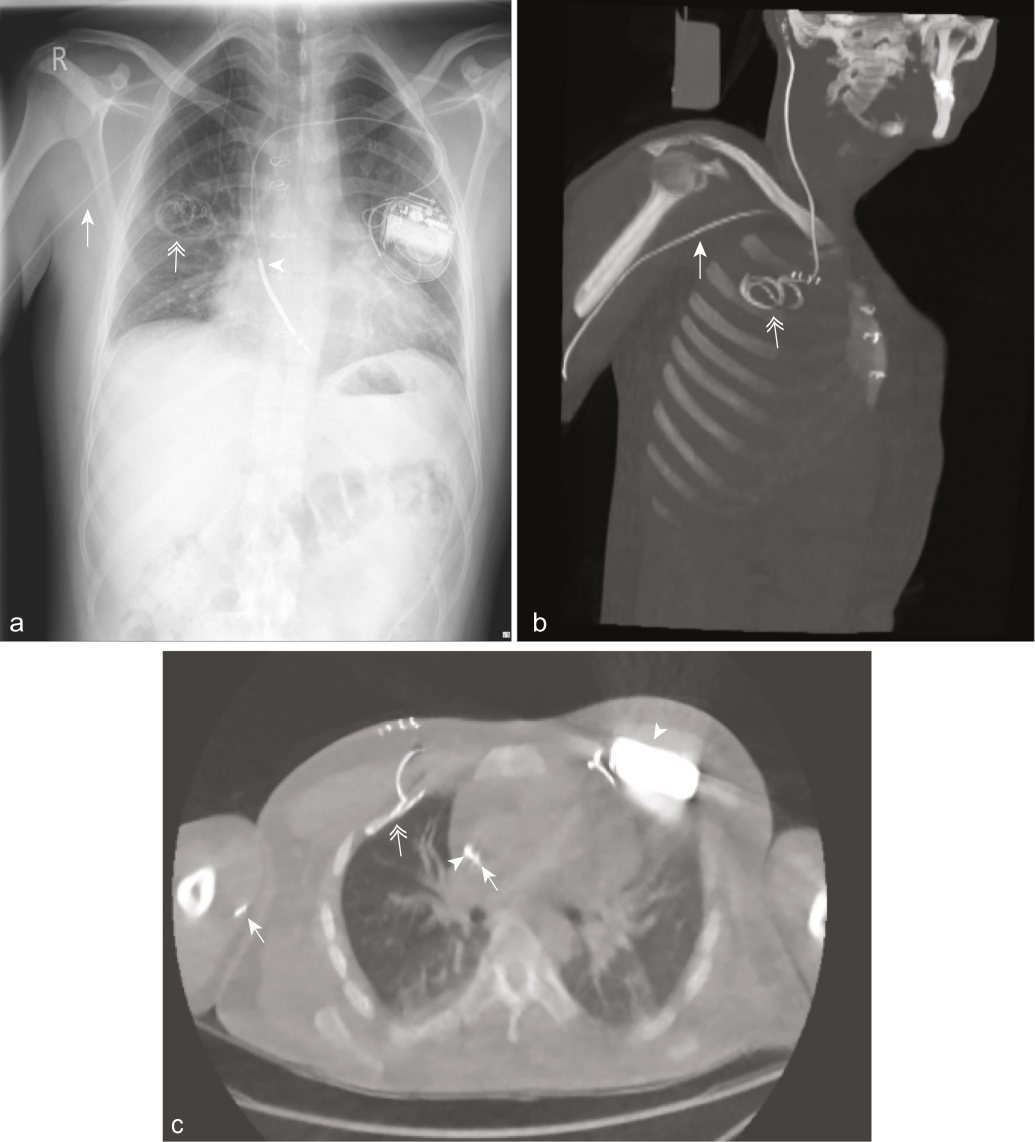
ULD CT with pure IR permits confident evaluation of shunt position postoperatively. Chest radiograph showing the distal end of a newly inserted ventriculopleural shunt (*double arrowhead*) in the mid-zone of the right hemithorax (**a**). ULD CT with pure IR multiplanar reconstructions clearly demonstrating the shunt course and precise anatomical localisation of the distal catheter tip in the pleural space overlying the right upper lobe (**b** and **c**). Skin clips and subcutaneous air related to the recent procedure are seen in the right anterior chest wall. On lung window, no pneumothorax or additional postoperative complication is demonstrated (**c**). Previous median sternotomy, left-sided single lead implantable cardioverter defibrillator (*arrowhead*) and right-sided peripherally inserted central catheter (*arrow*) are also demonstrated. *CT* Computed tomography, *IR* Iterative reconstruction, *ULD* Ultralow dose

Although the shunt tip was visible in 22/22 (100%) and 21/22 (95.5%) with ULD CT reconstructed with pure IR and PRSS, respectively, ULD CT reconstructed with pure IR showed superiority over PRSS in the demonstration of the exact distal shunt entry point to the peritoneum, pleura or atrium and permitted precise identification of the shunt tip anatomic location in all cases. This could not be evaluated with PRSS due to the inherent limitations of two-dimensional imaging (Table 3). To illustrate, following insertion of a new ventriculopleural shunt, ULD CT reconstructed with pure IR clearly demonstrated the pleural entry point and distal end of the catheter in satisfactory position in the pleural space overlying the right upper lobe (Fig. 5). This could not be evaluated on PRSS.

**Fig. 6:**
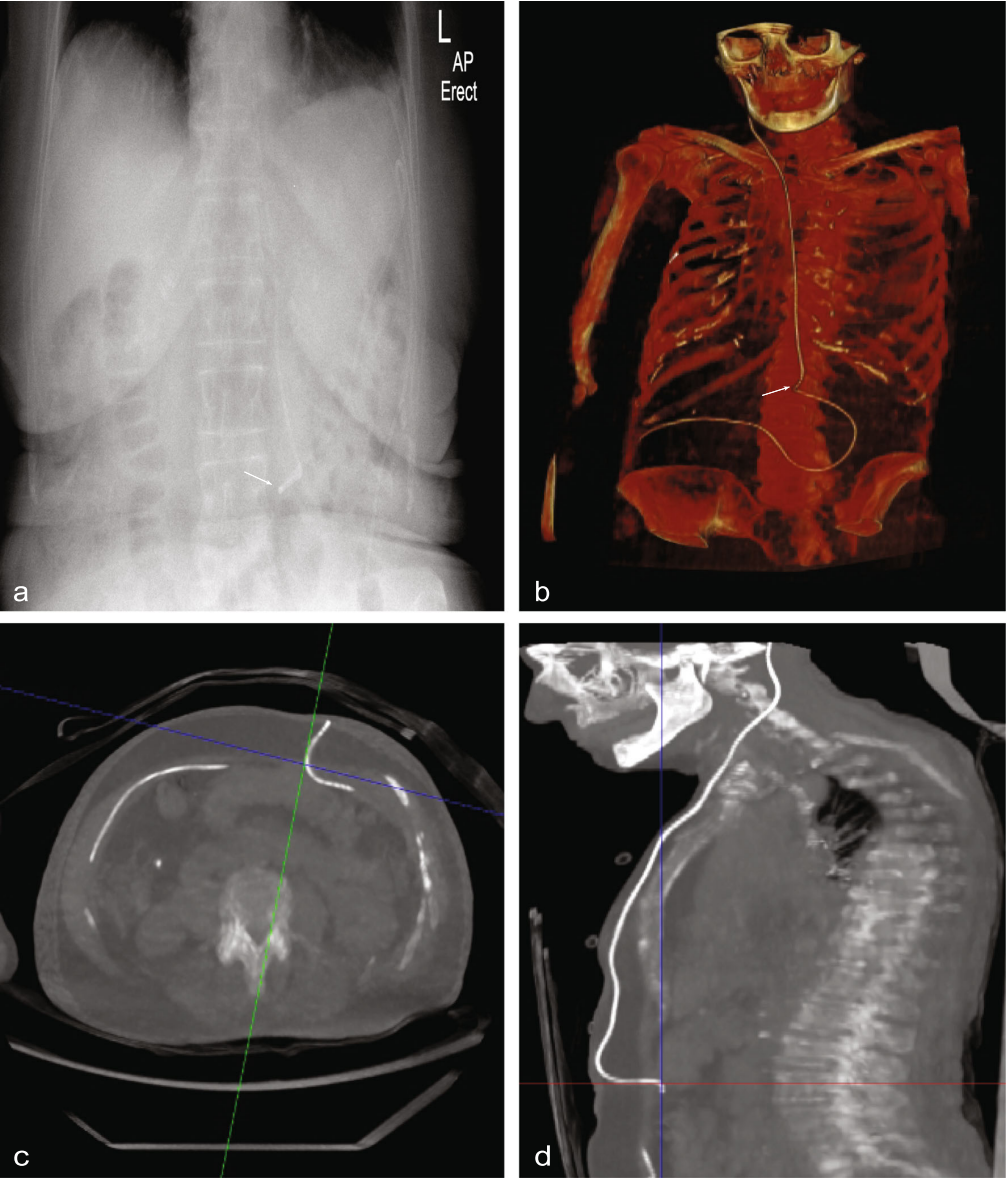
Specificity of ULD CT with pure IR for shunt evaluation. Abdominal radiograph portion of a plain radiographic shunt series (PRSS) demonstrating a kink in the shunt tube (*arrow* in **a**). ULD CT with pure IR three-dimensional reconstruction confirming the presence of a contour change in the shunt tubing at the corresponding level (*arrow* in **b**). Axial (42-mm reconstructed slice thickness) and coronal (34-mm reconstructed slice thickness) ULD CT images showing that the ‘kink’ seen on plain radiograph correlates with the peritoneal entry site of the shunt and was therefore a false positive (**c** and **d**). *CT* Computed tomography, *IR* Iterative reconstruction, *PRSS* Plain radiographic shunt series, *ULD* Ultralow dose

Both PRSS and ULD CT reconstructed with pure IR identified discontinuity in old shunt tubing in 4/18 (22.2%) patients. A possible kink in the shunt was observed in 3/18 (16.7%) patients on PRSS. On ULD CT reconstructed with pure IR, these were confirmed to be false positives with the area of concern corresponding to a change in the shunt contour at the peritoneal entry site in all cases (Fig. 6). No organ damage secondary to shunt complications was demonstrated on either ULD CT reconstructed with pure IR or PRSS. ULD-CT allowed confident exclusion of immediate postoperative intrathoracic complications such as pneumothorax (Fig. 5c).

#### Extra shunt-related performance of ULD CT reconstructed with pure IR

##### Brain

**Fig. 7:**
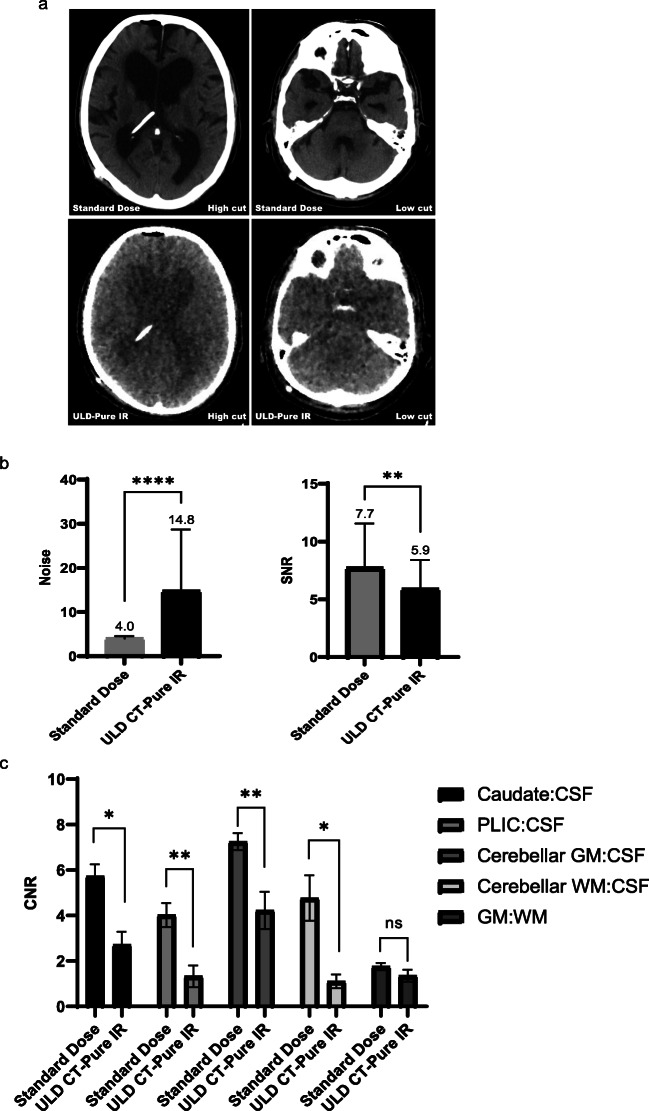
Increase in image noise for brain ULD CT with pure IR. **a** Two-level axial (3-mm slice thickness) computed tomography images acquired using standard dose and ULD in the same patient. Although discrimination of the ventricles is achieved on ULD CT with pure IR, subjective noise is high and image contrast is poor. **b** Image noise and SNRs in the standard dose and ULD CT with pure IR brain study from the same patients were compared. Difference in means for image noise and signal-to-noise ratio was assessed using a Wilcoxon matched-pairs signed rank test and paired samples *t*-test, respectively. Data represent the mean and standard deviation. A *p*-value < 0.05 was considered statistically significant. ****p < 0.001, **p = 0.004. **c** Comparison of contrast-to-noise ratios between the two techniques was performed using a paired samples *t*-test. Caudate: CSF *p = 0.021; PLIC: CSF **p = 0.004; cerebellar GM: CSF **p = 0.010. Cerebellar WM: CSF *p = 0.039. GM (caudate): WM (PLIC) *p* = 0.236. *CT* Computed tomography, *CSF* Cerebrospinal fluid, *IR* Iterative reconstruction, *PLIC* Posterior limb of internal capsule, *GM* Grey matter, *WM* White matter, *ULD* Ultralow dose

SNR and contrast-to-noise ratios were assessed in patients who in addition to the ULD study had a standard dose brain CT performed (120 kV, cerebrum/140 kV, skull base; 280 mA, cerebrum/330 mA, skull base; DLP 1021.4 ± 20.7 mGy*cm). In all patients who underwent ULD CT brain imaging (7/18, 38.9%), the intracranial shunt course and intraventricular catheter tip were clearly visible (Fig. 4b). However, the ULD images were subjectively nosier than the standard dose study limiting assessment of ventricular size and parenchymal images were non-diagnostic (Fig 7a). Corroborating the subjective appearances, in both the supra- and infratentorial compartments, ULD CT-Pure IR image noise (*p* < 0.001, Wilcoxon matched-pairs signed rank test), signal-to-noise (*p* = 0.004, paired sample *t*-test) and contrast-to-noise ratios were significantly inferior to the standard dose study (Fig. 7 b and c).

##### Thorax

One patient in addition to having a PRSS and ULD CT reconstructed with pure IR had a standard dose non-contrast-enhanced thoracic CT for investigation after an abnormal chest radiograph which permitted direct comparison of the two techniques (Supplementary Figs S2a,b). The standard dose thoracic CT (120 kV, Automatic Tube Current Modulation, DLP 304.4 mGy*cm) was reconstructed using hybrid IR. For both techniques, there was no significant difference in image noise and SNRs in the aorta (*p* = 0.110, noise; *p* = 0.363, SNR), lung (*p* = 0.442 and *p* = 0.431), muscle (*p* = 0.562 and *p* = 0.051), subcutaneous fat (*p* = 0.702 and *p* = 0.683) and liver (*p* = 0.135 and *p* = 0.253) (individual paired sample *t*-tests) (Supplementary Fig. S2c,d). Concordant with the objective noise findings, subjectively, ULD CT reconstructed with pure IR was comparable to the standard dose study, allowing confident pulmonary parenchymal evaluation, and in this patient clearly demonstrated consolidative change in both lungs and bilateral pleural effusions (Supplementary Fig. S2a,b). In the remaining cohort, clinically significant findings were seen in 3/18 (16.7%) patients with ULD CT reconstructed with pure IR; lung consolidation and pleural effusions in 2/18 patients (11.1%) and a single patient (1/18, 5.6%) had an indeterminate lung mass which was further evaluated to exclude malignancy (Supplementary Fig. S2e,f).

## Discussion

Here, we report the development and implementation of a whole-body ULD CT protocol for the evaluation of shunt failure that permits a 36% radiation dose reduction relative to conventional plain radiographic shunt series. Pala et al. [12] recently described a low-dose CT protocol for the evaluation of shunt dysfunction with an effective dose of 1.9 mSv. The achieved median effective dose with ULD CT in this study was 0.16 mSv which tentatively may permit a reduction in the cumulative radiation dose received by paediatric and adult patients. It is worth noting that the PRSS median effective dose in this study was 0.25 mSv while previous studies have quoted effective doses of 1.57 ± 0.60 mSv per PRSS study [5]. Therefore, the relative dose reduction effect size with ULD CT may have been underestimated in this study.

Dose reduction may result in increased image noise and reduced image quality when reconstructed using traditional filtered back projection [14]. To address this, we compared two iterative algorithms, hybrid IR and pure IR, demonstrating that at all anatomical levels in both the cadaveric model and patients, pure IR was superior with a significant reduction in objective image noise. Concordant with this reduction and objectively improved image quality, in the head and neck for both PRSS and ULD CT reconstructed with pure IR, shunt visualisation was rated as excellent. Interestingly, in the thoracic and abdominal cavities, shunt visualisation with ULD CT-Pure IR was significantly better than PRSS. Distal shunt failure due to malpositioning at time of insertion or secondary displacement, particularly in obese patients occurs in 10–30% of cases [15]. Therefore, clear visualisation of the distal shunt is essential representing a potential strength of ULD CT.

For all clinical parameters that could be directly compared between the two modalities including ability to visualise the entire imaged shunt course and shunt tip visibility, ULD CT reconstructed with pure IR was again superior with a perfect performance in each tested category. Moreover, in contrast to the two-dimensional properties of PRSS, ULD CT due to its multiplanar capabilities allowed accurate three-dimensional localisation of the shunt tip and could identify the entry point into the peritoneum, pleural or atrium in all cases. This property may be helpful when assessing the position of newly inserted shunts. In this study, the entire imaged shunt course and location of the distal shunt tip in all newly inserted catheters were clearly visualised using ULD CT. In addition, in contrast to PRSS, ULD CT allowed confident exclusion of immediate post-procedural-related complications as in the illustrative case of the ventriculopleural shunt.

Visualisation of tissues and organs contiguous with the shunt on ULD CT-Pure IR was rated as satisfactory to excellent at all anatomical levels. This could not be assessed on PRSS. Although not seen on this study, it is conceivable that this ability will permit evaluation of distal catheter-related complications such as intraperitoneal and extraperitoneal fluid collections, bowel perforation and large pleural effusions in patients with ventriculopleural shunts [16, 17]. This is of clinical significance as it has been shown that distal catheter-related complications in the thoracoabdominal cavities can precede and predict the development of hydrocephalus [16].

The sensitivity of ULD CT relative to the standard PRSS for the identification of shunt discontinuity was 100%. Inconclusive appearances on PRSS are known to result in repeat and/or additional imaging to clarify findings further contributing to increased patient dose [10]. In keeping with these findings, ‘kinks’ in the distal catheter were reported on PRSS in three patients in this study which were subsequently shown to be false positives on ULD CT. This improved specificity with ULD CT may further facilitate dose reductions.

Proximal catheter obstruction is the most common cause of shunt malfunction; therefore, precise assessment of the proximal shunt tubing is crucial [18]. In those patients that had ULD CT imaging of the brain, intracranial shunt position was easily assessed in all cases. However, when objectively compared to conventional dose CT brain, subjective and objective image noise was significantly greater in the low-dose study with a corresponding reduction in both signal and contrast-to-noise ratios. Therefore, although fit for purpose in shunt evaluation, ULD CT Brain only permits a limited evaluation of ventricular size, and it is not suitable for parenchymal evaluation or exclusion of acute pathologies including stroke or haemorrhage, and a standard dose study is also recommended. Further work is required to develop a low-dose protocol for brain that remains diagnostically acceptable for non-shunt-related pathology.

In terms of diagnostic accuracy, it has been shown that ULD thoracic CT reconstructed using iterative algorithms can produce images equivalent to full-dose studies [19, 20]. Concordant with these findings, image noise and signal-to-noise ratios on thoracic ULD CT reconstructed with pure IR were comparable to the full dose study. In febrile patients in whom shunt infection is queried, lung parenchymal assessment on ULD CT may help identify an alternative or additional source of sepsis such as aspiration. Moreover, the ability to detect clinically significant incidental findings on ULD CT is a significant advantage of this technique as evidenced by the case of the lung mass detected on ULD CT reconstructed with pure IR which was poorly defined on plain radiograph.

Limitations of our study include a small sample size and absence of a shunt complication in the current implanted shunt system in any patient. In addition, subjective image quality was assessed in consensus representing a potential methodological shortcoming. The influence of patient body composition on image quality and radiation dose was not specifically addressed. Alterations in body habitus in shunted patients including elevated body mass index and distorted anatomy often secondary to spinal deformity are common. These factors are well-known to contribute to poor image quality and increased radiation doses, the latter of which is explained in part by the need for repeat PRSS exposures. Future studies may look at the performance of ULD CT in these patient cohorts. A disadvantage of the technique is the longer reconstruction time associated with pure IR relative to FBP or hybrid IR. To illustrate, the reconstruction times using pure IR and hybrid IR in one randomly chosen patient from this study were 85 min and 30 s, respectively. Newer reconstruction methods incorporating deep-learning approaches may allow comparable low-dose high-quality images to be obtained in a much shorter timeframe in the near future. In addition, even though a low-dose study, the protocol involves the use of ionising radiation. As an alternative, in an attempt to minimise radiation exposure, some institutions have adopted magnetic resonance imaging (MRI) as the initial modality of choice in shunt failure [21]. However, possible changes in the valve pressure settings with programmable valves after being placed in the static magnetic field is a potential shortcoming of this approach [22, 23]. An advantage of ULD CT over MRI is the ability to assess the entire shunt course and extra-shunt tissues with a high spatial resolution. In addition, as in most acute settings, CT scanning in these patients is more straightforward than MRI being completed faster and is safer especially if there are devices for support or monitoring *in situ*, and if there is a requirement for staff to accompany the patient into the imaging suite.

With diagnostic superiority over PRSS at a significantly reduced ED, these results support the replacement of standard PRSS with whole-body ULD CT in the investigation of suspected shunt failure in paediatric and adult patients. It is envisaged that the phantom and cadaveric methodology presented herein may facilitate the development of low-dose imaging protocols at other institutions. By facilitating conventional CT imaging of the brain and low-dose CT imaging of the entire shunt in one location, the requirement for additional investigations and patient transfers is eliminated.

## Supplementary Information

**Additional file 1: Supplementary Table 1.** Anatomical Substructures Assessed for Objective Image Noise.

**Additional file 2: Supplementary Figure 1.** Superior 2D Shunt Visualisation with ULD CT-Pure IR Comparison of the median Likert scores for ULD CT-Pure IR and PRSS using Wilcoxon matched-pairs signed rank tests. Data represent the median, interquartile range, and minimum and maximum values. In the head and neck, PRSS and ULD CT-Pure IR were comparable with no significant difference observed (*p* = ns, not significant). In the thorax and abdomen however, visualisation quality was significantly better with ULD CT-Pure IR (**p = 0.004 and *p = 0.031 respectively).

**Additional file 3: Supplementary Figure 2.** ULD CT-Pure IR Permits Evaluation of Lung Parenchyma (a and b) ULD CT-Pure IR (a) and standard dose (b) coronal thoracic CT images demonstrating comparable subjective image quality. Bilateral consolidative change and pleural effusions are clearly appreciated on both studies (c and d). Objective noise (c) and signal to noise ratios (d) for standard and ULD CT-Pure IR images of the thorax and upper abdomen were calculated in the aorta, lung, paraspinal muscle, subcutaneous fat, and liver at the same anatomical level for both studies in a single patient. Data represent the mean and standard deviation. Difference in means was assessed using paired sample t-tests. A *p*-value < 0.05 was considered statistically significant. *SNR* Signal to Noise Ratio, *NS* Not significant (e and f) (e) Anteroposterior chest radiograph and corresponding coronal ULD CT (f) showing a mass lesion *(arrow)* in the right upper lobe on CT which is poorly appreciated on the plain radiograph. All CT images were reconstructed to a 3 mm slice thickness and visualised using lung windows (window width; 1500 HU, window level; -500 HU).

## Data Availability

All data generated or analysed during this study are included in this published article and its supplementary information files.

## Abbreviations

ASIR: Adaptive statistical iterative reconstruction
CSF: Cerebrospinal fluid
CT: Computed tomography
DLP: Dose length product
ED: Effective dose
FBP: Filtered back projection
IR: Iterative reconstruction
MBIR: Model-based IR
MRI: Magnetic resonance imaging
PRSS: Plain radiographic shunt series
ROI: Region of interest
SNR: Signal-to-noise ratio
ULD: Ultralow dose
