# Supplementary material for: Development and implementation of an ultralow-dose CT protocol for the assessment of cerebrospinal shunts in adult hydrocephalus
Source: Eur Radiol Exp. 2021 Jun 28;5:26. doi: 10.1186/s41747-021-00222-4 (PMC8236421; doi:10.1186/s41747-021-00222-4)
Supplement: Supplementary file 1 [file 41747_2021_222_MOESM1_ESM.docx]

**Anatomical Cavity**	**Anatomical Cavity Sub-level**
Head	Level of highest ventricle
	Right temporal lobe / middle cranial fossa
	Right pons
Neck (level of thyroid cartilage in line with right shoulder)	Right sternocleidomastoid muscle
	S/C fat right neck
Thorax (level of thoracic aortic arch)	Right pectoralis major
	S/C fat right anterior chest wall
	Aorta
	Right rectus abdominis muscle
Abdomen (level of porta hepatis)	S/C fat right anterior abdominal wall
	Liver
Pelvis (level of right iliac crest)	Right rectus abdominis muscle
	S/C fat right inferior abdominal wall

S/C subcutaneous
