# Supplementary material for: Development and implementation of an ultralow-dose CT protocol for the assessment of cerebrospinal shunts in adult hydrocephalus
Source: Eur Radiol Exp. 2021 Jun 28;5:26. doi: 10.1186/s41747-021-00222-4 (PMC8236421; doi:10.1186/s41747-021-00222-4)

Supplementary Fig. 1

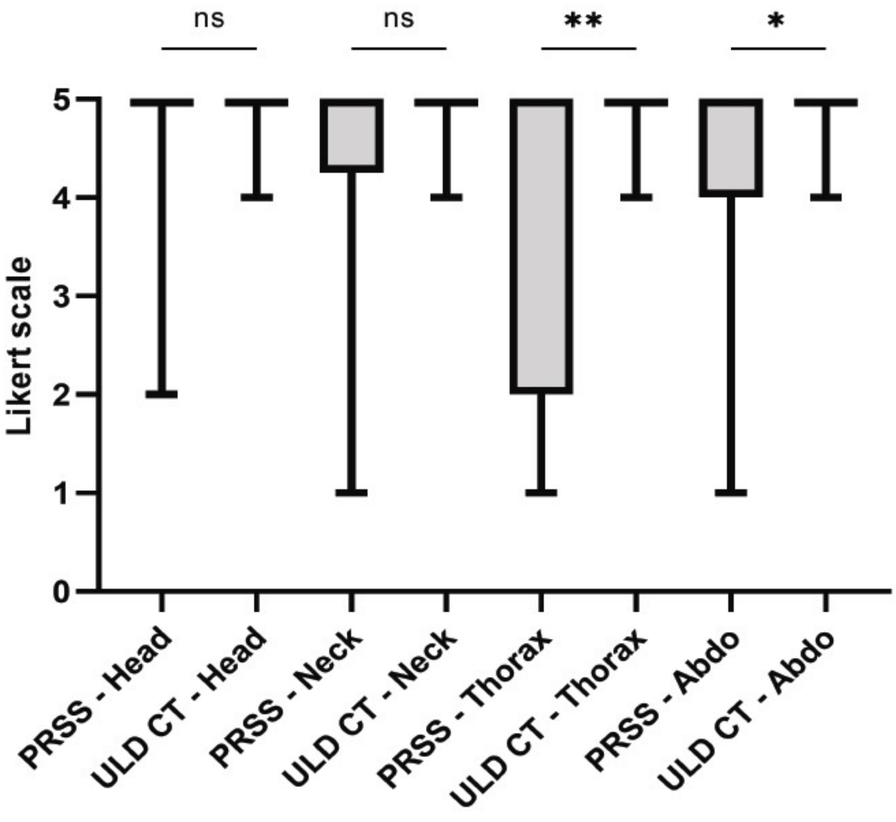

Supplement: Supplementary file 2 [file 41747_2021_222_MOESM2_ESM.docx]
